# Identification of a novel immune-related gene signature for prognosis and the tumor microenvironment in patients with uveal melanoma combining single-cell and bulk sequencing data

**DOI:** 10.3389/fimmu.2023.1099071

**Published:** 2023-01-30

**Authors:** Wanpeng Wang, Han Zhao, Sha Wang

**Affiliations:** ^1^ Eye Center of Xiangya Hospital, Central South University, Changsha, China; ^2^ Hunan Key Laboratory of Ophthalmology, Hunan, Changsha, China; ^3^ National Clinical Research Center for Geriatric Disorders, Xiangya Hospital, Changsha, China; ^4^ Department of Ophthalmology, Eye, Ear, Nose, and Throat Hospital of Fudan University, Shanghai, China

**Keywords:** uveal melanoma, immune-related gene, prognostic signature, immune checkpoint, tumor microenvironment

## Abstract

**Introduction:**

Uveal melanoma (UVM) is the most invasive intraocular malignancy in adults with a poor prognosis. Growing evidence revealed that immune-related gene is related to tumorigenesis and prognosis. This study aimed to construct an immune-related prognostic signature for UVM and clarify the molecular and immune classification.

**Methods:**

Based on The Cancer Genome Atlas (TCGA) database, single-sample gene set enrichment (ssGSEA) and hierarchical clustering analysis were performed to identify the immune infiltration pattern of UVM and classify patients into two immunity clusters. Then, we proposed univariate and multivariate Cox regression analysis to identify immune-related genes that related to overall survival (OS) and validated in the Gene Expression Omnibus (GEO) external validation cohort. The molecular and immune classification in the immune-related gene prognostic signature defined subgroups were analyzed.

**Results:**

The immune-related gene prognostic signature was constructed based on S100A13, MMP9, and SEMA3B genes. The prognostic value of this risk model was validated in three bulk RNA sequencing datasets and one single-cell sequencing dataset. Patients in the low-risk group had better OS than those in the high-risk group. The receiver-operating characteristic (ROC) analysis revealed its strong predictive ability for UVM patients. Lower expression of immune checkpoint genes was presented in the low-risk group. Functional studies showed that S100A13 knockdown via siRNA inhibited UVM cell proliferation, migration, and invasion *in vitro*, with the increased expression of reactive oxygen species (ROS) related markers in UVM cell lines.

**Discussion:**

The immune-related gene prognostic signature is an independent predictive factor for the survival of patients with UVM and provides new information about cancer immunotherapy in UVM.

## 1 Introduction

Uveal melanoma (UVM) is one of the most common malignant ocular tumors in adults (1). The age-adjusted incidence rate of UVM in the United States was 5.2 cases per million individuals per year (2). The three types of UVM cells are spindle, epithelioid, and mixed. The choroid accounts for approximately the majority of tumor instances, with the remaining cases arising from the ciliary body and iris. Because iris melanomas (IMs) are typically visible from the outside of the eye and are diagnosed earlier than other types of melanomas, they typically have a better prognosis than the others (3). UVM mainly originates from melanocytes in the choroid, ciliary body, and iris. UVM is a highly heterogeneous tumor. Chromosomal alterations and genetic mutations are suspected to take part in the initiation and development of UVM (4, 5). Currently, the main treatment for UVM is enucleation, resection, and radiation therapies. However, the prognosis of UVM patients was still poor due to the resistance, recurrence, and metastasis. More than 50% of patients with primary UVM will eventually develop distant metastasis, up to 90% of individuals have liver involvement and have a median survival of fewer than 6 months (6–8). However, early detection and surgical treatment of metastasis UVM could enhance the progression-free survival and overall survival (OS) in UVM patients (9–12). Therefore, identifying a novel prognosis biomarker is of great importance to improve the survival outcomes of patients with UVM.

Compared with traditional therapies, targeted therapies, and immunotherapy, such as immune checkpoint blockade (ICB), vaccination, and adoptive T-cell therapy, have shown significant benefits in multiple types of human cancers (13, 14). Despite the effectiveness of immunotherapy in advanced cutaneous melanoma, the effectiveness of UVM patients is still unsatisfactory. The major limitation of targeted therapies or immunotherapy is the low clinical response rate, approximately 0 to 5% (15). The tumor immune microenvironment and immune cell infiltration (ICI) that influence the prognosis of UVM patients have been extensively investigated in the past few decades. Accumulating evidence reveals that the tumor immune microenvironment plays an important role in cancer development and progression and response to treatment (16). Korn et al. found that T helper cell 17 (Th17) had strong anti-tumor activity *via* secreted interleukin-21 (IL-21) (17). According to a multicenter phase II study, IL-21 had a certain curative effect on metastatic melanoma (18). Moreover, a clinical trial proved the antitumor activity of programmed cell death ligand 1/programmed cell death 1(PD-L1/PD-1) signaling blocking in advanced melanoma (19). In addition, antibodies of cytotoxic T-lymphocyte-associated protein 4 (CTLA-4), such as ipilimumab and tremelimumab, were confirmed useful in metastatic melanoma. Snyder et al. reported that genetic basis was closely related to the clinical response rate of anti-CTLA-4 therapy for metastatic melanoma (20). Another immune checkpoint is lymphocyte-activation gene-3 (LAG3) was present on the surface of T cells, NK cells, and plasmacytoid dendritic cells and it had a positive association with other immune checkpoints and immune modulators (21, 22).

With the continual improvement in understanding the immune system in the tumor, it can help us to find a biomarker that predicts patients’ survival outcomes, and it can also help clinicians to identify patients who are responders and non-responders to ICB treatment (23, 24). In our previous studies, we explored the intratumoral immune infiltration landscape in UVM and constructed an ICI score to predict the prognosis of patients with UVM (25). Recently, several studies have reported some prognostic signatures based on the immune-related gene to predict the survival outcome of various cancers, such as ovarian carcinoma (26), osteosarcoma (27), and Glioblastoma (28). However, the role of immune-related genes in the progression and development of UVM needed to be elucidated.

To better understand the immune-related gene in UVM, in this study, we conducted a comprehensive analysis in UVM cohorts from The Cancer Genome Atlas (TCGA) and explored the role of the immune-related gene on the prognosis of UVM patients. We used single-sample gene set enrichment analysis (ssGSEA) to classify UVM patients into Immunity_H and Immunity_L clusters and analyzed them with ESTIMATE and CIBERSORT algorithms. Then, we screened the differentially expressed genes (DEGs) between the Immunity_H and Immunity_L clusters. Subsequently, we selected the intersection gene between the immune-related gene and DEGs and constructed an immune-related gene prognostic signature by using univariate Cox regression, least absolute contraction, and selection operator (LASSO) analyses, and support vector machine-recursive feature elimination (SVM-RFE) algorithm. In addition, we analyzed the prognostic values of the risk model by using Kaplan-Meier survival analysis and Cox analysis, which was further validated in the Gene Expression Omnibus (GEO). GSE139829, the single-cell sequencing data set from UVM, was chosen for single-cell sequencing analysis due to its clinical data and reasonably large sample size. We validated the expression levels of three immune-related genes in the normal retinal pigmental epithelial cell line (ARPE-19) and UVM cell line (C918). Finally, we used cell experiment to verify the role of the most significant gene in this signature, S100A13. After a series analysis, we identified an immune-related gene prognostic signature that could function as an effective and independent prognostic of patients with UVM.

## 2 Materials and methods

### 2.1 Transcriptome data collection and processing

For the TCGA cohort, clinical features, RNA-seq expression data [fragments per kilobase million (FPKM) value], and somatic mutation data were downloaded from the TCGA database (https://cancergenome.nih.gov/). For the GEO cohort, clinical features and RNA-seq expression data were obtained from the GEO database (https://www.ncbi.nlm.nih.gov/geo/). UVM samples from GSE22138 (platform GPL570, Affymetrix Human Genome U133 Plus 2.0 Array), GSE44295 (platform GPL6883, Illumina HumanRef-8 v3.0 expression beadchip), and GSE84976 (platform GPL10558, Illumina HumanHT-12 V4.0 expression beadchip) were used as the matched validation datasets, included 63, 57, and 28 UVM patients, respectively. Then, we extracted and constructed the gene expression matrix from the TCGA-UVM, GSE22138, GSE44295, and GSE84976 datasets by using Strawberry Perl (version 5.32.02). The clinical and pathological characteristics of each patient in the CGA-UVM, GSE22138, GSE44295, and GSE84976 are summarized in **Supplementary Table 1**.

### 2.2 Single-cell sequencing data collection and processing

The UVM single-cell data set GSE139829 was downloaded. The following were used as inclusion criteria (1): histologically verified primary UVM and no metastases; (2) pathology cell type was mixed. The next step is data quality control. Cells with fewer than 20% mitochondrial genes, cells with more than 200 genes overall, and genes with expression ranging from 200 to 7000 and expressed in at least six cells were all kept. The “FindVariableFeatures” R package was used to identify the 3000 genes that were the most variable, and the “ScaleData” R package was used to scale the data. Then, the UMAP method was used to lower the dimension of the data, and the “KNN” method was used to execute cell clustering with a resolution of 1.0. Cells were then annotated by different cell surface markers. Based on Single-cell sequencing data, the “CellChat” R package [27]was used to identify, display, and evaluate intercellular communication across several cell types. CellChat was also used to distinguish signaling pathways.

### 2.3 Clustering for uveal melanoma data

We applied ssGSEA to group UVM transcriptome data from the TCGA. We obtained a set of immune-related cells and types, including immune cell types, immune-related pathways, and immune-related functions[28]. Based on the 29 immune data sets, the infiltration level of different immune cells in each UVM sample was quantified by using Gene Set Variation Analysis (GSVA) R package. According to the results of ssGSEA, Patients with HNSCC were stratified into two clusters Immunity_H and Immunity_L clusters by using the “hclust” R package.

### 2.4 Verification of the effective immune grouping

ESTIMATE algorithm was designed to calculate the levels of the immune and stromal cells, namely Immune Score and Stromal Score, which represent the abundance of immune and stromal components, respectively. The ESTIMATE Score, Immune Score, and Stromal Score of each UVM sample in two clusters of the TCGA database were calculated by using the “estimate” R package to verify the effect of the ssGSEA grouping. The hierarchical clustering heatmap of UVM samples with ESTIMATE score was executed. Infiltration levels of distinct immune cells in UVM samples were determined by using the CIBERSORT deconvolution algorithm[29]. Then, we used the CIBERSORT algorithm to investigate the proportion of 22 types of immune cells in two clusters of the TCGA database using the “CIBERSORT” R package to validate the effectuality of ssGSEA grouping again. Besides, we analyzed the expression of the human leukocyte antigen (HLA) family between the Immunity_H and Immunity_L clusters using the “ggpubr” R package.

### 2.5 Identification of immune-related gene in uveal melanoma

Based on the above-mentioned clusters, TCGA-UVM data was divided into Immunity_H and Immunity_L clusters. According to the criteria of p <0.05 and |log2FC| > 2, we used the “edgeR” and “limma” R package to obtain the DEGs between these two clusters. To investigate the immune-related prognostic signature of UVM, 2498 immune-related genes were obtained from the ImmPort database (https://www.immport.org)[30]. The ImmPort database contains a series of immune-related genes such as the genes relating to the macrophages, natural killer cell cytotoxicity, B cell antigen receptor signaling pathway, and T cell receptor signaling pathway. Then, the Venn diagram identified immune-related genes from the above-mentioned results.

### 2.6 Functional enrichment analysis

To predict the potential function of intersection genes, we performed gene set enrichment analysis between the Immunity_H and Immunity_L clusters of the TCGA database to determine the Kyoto Encyclopedia of Genes and Genomes (KEGG) pathway. All the analyses were performed by “clusterprofiler” and “enrichplot” R packages. The p < 0.05 and FDR < 0.05 were considered to be statistically significant.

### 2.7 Development of the immune-related gene prognostic signature for uveal melanoma

According to the clinical data of UVM samples in the TCGA, univariate Cox regression analysis was used to screen immune-related gene significant correlation to the survival of UVM patients by using the “survival” R package. Then, we used LASSO regression analysis to identify genes most correlated with the OS of UVM samples by using the “glmnet” R package. 1000-round cross-validation for penalty parameter selection was performed to minimize overfitting and using the SVM-RFE technique to get the variable’s lambda with the least classification error. Based on the coefficients from the multivariate Cox regression analysis and expression level of prognostic-related immune genes, we constructed the prognostic signature for prognostic outcome prediction of UVM. The formula is as follows:


(1) $$\text{Risk score (patients) }=\sum_{i=1}^{n}Expression_{Genei}\;\times \;Coefficient_{Genei}$$

Here, “n” represents the number of prognostic genes; “i” is the serial number of each gene. Relying on the median value of risk score (“Survminer” R package), the UVM patients were stratified into high-risk and low-risk groups. The Kaplan-Meier survival analysis was performed to assess the survival rate and median survival for high-risk and low-risk groups. The log-rank test was used to evaluate the difference in survival between these two groups. The time-dependent receiver-operating characteristic (ROC) was used to calculate the specificity and sensitivity of the risk model using the “timeROC” R package. Further, univariate Cox and multivariate Cox regression analyses were used to assessing the independence of the prognostic signature of OS of UVM patients from some key clinical factors such as gender, age, and metastasis status using the “survival” R package.

### 2.8 Verification of the immune-related gene prognostic signature for uveal melanoma

To investigate the stability and repeatability of the multi-gene prognostic model, we used GSE22138, GSE44295, and GSE84976 datasets as independent validation cohorts and calculated the risk score of each patient. The Kaplan-Meier survival analysis and log-rank test were performed to evaluate the differences in survival rates between the two groups. The ROC curve was implemented to show the predictive ability of prognostic signatures in the validation cohorts.

### 2.9 Construction and verification of nomogram

The nomogram was an effective method to predict the survival of UVM patients by transferring complex statistical models into a contour map. The risk score, age, gender, primary tumor site, and metastasis status were used to build the nomogram based on the immune-related gene prognostic signature using “rms” and “survival” R packages. Meanwhile, the calibration curve was used to evaluate the predicted probability of the nomogram in differentiating between patient groups.

### 2.10 Cell culture and transfection

The adult retinal pigment epithelial cell line-19 (ARPE-19) and the human invasive UVM cell line (C918) were purchased from Procell Life Science&Technology Co., Ltd (Wuhan, Hubei, China). ARPE-19 cell was cultured in Dulbecco’s Modified Eagle’s Medium/Nutrient Mixture F12 Ham’s Liquid media (DMEM/F-12; Cytiva/Global Life Sciences Solutions, Marlborough, MA) containing 10% fetal bovine serum (FBS; Gibco, Carlsbad, CA, USA), along with 100 U/mL penicillin and streptomycin (Gibco, Carlsbad, CA, USA). C918 cell was cultured in Roswell Park Memorial Institute 1640 liquid media (RPMI1640; Gibco, Carlsbad, CA, USA) containing 10% fetal bovine serum (FBS; Gibco, Carlsbad, CA, USA), along with 100 U/mL penicillin and streptomycin (Gibco, Carlsbad, CA, USA). Cells were maintained in an incubator (Thermo Fisher Scientific, Waltham, MA) at 37°C, with 95% humidity, and 5% CO2. Cell culture plates, round coverslips, and centrifuge tubes were obtained from (NEST Biotechnology; Wuxi, China). C918 cells were transfected with the generated small interfering RNAs (RiboBio; Guangzhou, China) targeting gene S100A13 and its control siRNAs, according to the manufacturer’s procedure. The siRNA sequences for the gene S100A13 were ACTCGGAGCTCAAGTTCAA.

### 2.11 Quantitative real−time polymerase chain reaction

Total cellular RNA of ARPE-19 and C918 was extracted and purified by using the SteadyPure Quick RNA Extraction kit (ACCURATE BIOTECHNOLOGY(HUNAN)CO.,LTD, Changsha China; AG21023) according to the manufacturer’s instructions. cDNA was synthesized by using an Evo M-MLV Mix Kit with gDNA Clean for qPCR (ACCURATE BIOTECHNOLOGY(HUNAN)CO.,LTD, Changsha China; AG11728). Real-time PCR was performed using the SYBR^®^ Green qPCR Kit (ACCURATE BIOTECHNOLOGY(HUNAN)CO.,LTD, Changsha China; AG11701). Relative expression of the target gene was calculated by using the 2−ΔΔCT method.

Primers for qRT-PCR include:

h-MMP9-F, AGTCCACCCTTGTGCTCTTCCC,

h-MMP9-R, TCTCTGCCACCCGAGTGTAACC;

h-SEMA3B-F, AGGAAGGATAGAGGATGGCAAGGG,

h-SEMA3B-R, AGGCTGCGAAAGATGGTAAAGTCTC;

h-S100A13-F, TCCTAATGGCAGCAGAACCACTGA,

h-S100A13-R, TTCTTCCTGATTTCCTTGGCCAGC;

h-KEAP1-F, AACGGTGCTGTCATGTACCA,

h-KEAP1-R, GGCAGTGGGACAGGTTGAA;

h-NRF2-F, TCCAGTCAGAAACCAGTGGAT,

h-NRF2-R, GAATGTCTGCGCCAAAAGCTG;

h-HO-1-F, TTCAAGCAGCTCTACCGCTC,

h-HO-1-R, GAACGCAGTCTTGGCCTCTT;

h-NQO1-F, TATCCTGCCGAGTCTGTTCTG,

h-NQO1-R, AACTGGAATATCACAAGGTCTGC;

h-β-actin-F, GAAGATCAAGATCATTGCTCCT,

h-β-actin-R, TACTCCTGCTTGCTGATCCA.

### 2.12 Western blotting analysis

The ARPE-19 and C918 cell was lysed by using M-PER mammalian protein extraction reagent (Thermo Fisher Scientific, Waltham, MA) containing protease inhibitors (Sigma-Aldrich, St Louis, MO, USA), and total proteins were extracted from lysis buffer. After quantifying the concentrations of total proteins by using a BCA kit (Sangon Biotech, Shanghai, China) equal amounts of protein were used to perform western blotting. Protein samples were separated on sodium dodecyl sulfate-polyacrylamide gel and transferred to polyvinylidene difluoride membranes (Merck-Millipore, Billerica, MA, USA). After blocking by using 5% milk for 1 hour, the polyvinylidene difluoride membranes were incubated overnight with primary antibodies at 4°C. Primary antibodies used in the study were listed as follows: anti-MMP9 (Proteintech, 10375-2-AP, 1:1000), anti-S100A13 (Proteintech, 14987-1-AP, 1:1000), anti-SEMA3B (ABclonal, A7004, 1:1000), anti-HO-1 (Arigo, ARG43341, 1:1000), and anti-NQO-1 (Arigo, ARG43340, 1:1000). After incubation with HRP-goat anti-rabbit (Elabscience, E-AB-1003, 1:3000), immunoblots were visualized by chemiluminescence reagent (Merck-Millipore, Billerica, MA, USA) and analyzed by Image J software v1.49.

### 2.13 Cells viability assay

The vitality of the C918 cells was assessed using the Cell Counting Kit-8 (CCK-8) technique. After adhering, the cells were planted on a 96-well cell culture plate and then underwent siRNA transfection. CCK-8 solution (UElandy Inc, Suzhou, China; C6005L) was poured into each well of the plate 24 hours, 48 hours, and 72 hours after siRNA transfection. The dish was then placed in the cell culture incubator, which was dark. Finally, a microplate reader was used to measure the absorbance at 450 nm wavelength (Tecan, Männedorf, Switzerland). For the live/dead labeling, samples were incubated for 30 minutes at 37°C with calcein acetoxymethyl ester (Calcein AM) and propidium Iodide (PI) in PBS before imaging.

### 2.14 Cells proliferation assay

According to the manufacturer’s instructions, the YF^®^ 488 Click-iT EdU Kit (UElandy Inc, Suzhou, China; C6015L) was used to assess the proliferation of C918 cells. Shortly after removing the media, 10 M of EdU solution was added, and the mixture was cultured for two hours at 37°C. C918 cells were fixed with 4% paraformaldehyde. After permeabilization, all samples were incubated with the Hoechst 33342. Stained C918 cells were washed three times with PBS and counted under a fluorescent microscope (Leica Microsystems, Wetzlar, Germany).

### 2.15 Transwell assay

The cellular migration was assessed using a transwell chamber with a pore size of 8.0 μM (NEST Biotechnology, Wuxi, China). Upon transfection, C918 cells were seeded in low serum (1% FBS) media in the top chamber with Matrigel solution (BD Biosciences, San Diego, CA, USA). A complete medium was added to the lower chamber. The chambers were incubated for 24 hours. The cells on the bottom side of the transwell membrane were fixed with 4% paraformaldehyde and stained with crystal violet after the cells on the top side of the membrane were scraped away, and counted under a light microscope (Leica Microsystems, Wetzlar, Germany).

### 2.16 Scratch wound healing assay

Upon transfection, the C918 cells were cultured on a 6-well plate until they were 90–100% confluent. One line of the C918 was scraped in each culture well using a sterile plastic pipette tip. To eliminate cellular debris, the cells were washed twice with PBS. The scratch wounds were photographed under a microscope (Leica Microsystems, Wetzlar, Germany) at 0 and 24 hours, and the percentage of the wound closure area was used to determine cell migration.

### 2.17 Reactive oxygen species detection

Upon transfection, the C918 cells were cultured on a 96-well plate. After discarding the supernatants, H2DCF-DA (10µM; Dojindo; Kumamoto, Japan) or dihydroethidum (DHE, 10µM; Beyotime, Shanghai, China) was added to stain the sample for 30 minutes at 37°C. Following PBS washes, cells were fixed with 4% paraformaldehyde for 15 minutes and then examined under a fluorescence microscope (Leica Microsystems, Wetzlar, Germany).

### 2.18 Human UVM samples and immunohistochemistry

The paraffin specimens of UVM tissue and para-carcinoma tissue samples were collected. The sample of tissue was sectioned into 5 μm thick sections and set on glass slides that were dried overnight at 37°C, dewaxed, and rehydrated. After pre-incubating with 3% bovine serum albumin, sections were incubated with the primary antibody specific for anti-S100A13 (Proteintech, 14987-1-AP, 1:50). At the same time, negative controls were conducted by incubating a slice with PBS and no primary antibody. Then, the slides were incubated with secondary antibody and viewed under a light microscope (Eclipse ci; Nikon, Tokyo, Japan).

### 2.19 Statistical analysis

All statistical analyses were conducted using the R software (version 4.0.4, 64-bit; https://www.r-project.org/) and its appropriate packages. The Kaplan-Meier analysis and log-rank test were applied to assess survival and compare the difference in survival between clusters as well as risk groups. Two-tailed p < 0.05 was regarded statistically significant.

## 3 Results

### 3.1 Construction and validation of uveal melanoma clustering

**Figure 1:**
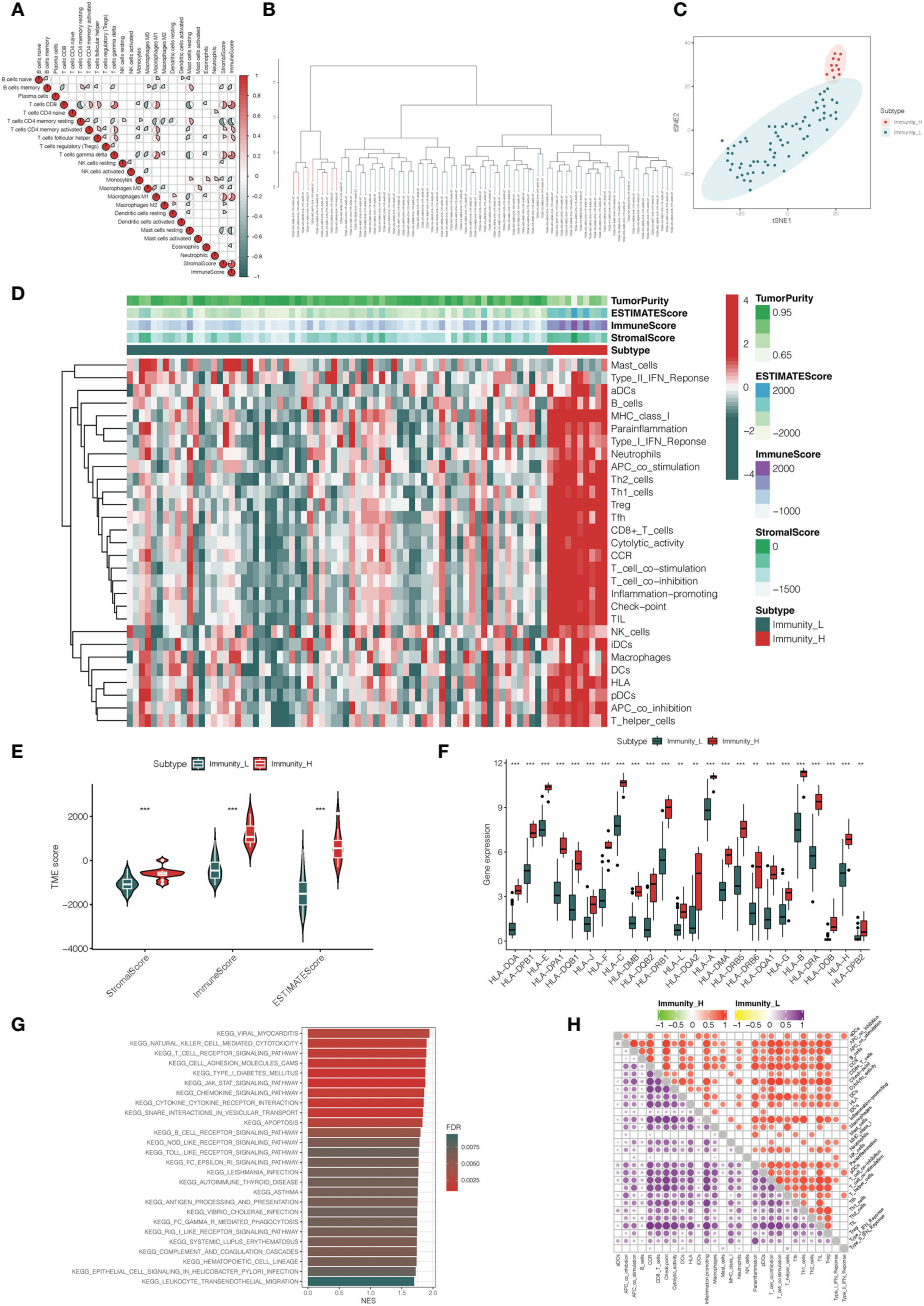
Construction and verification of uveal melanoma clustering. **(A)** A correlation matrix serves as a representation for all 22 invading immune cells. While certain immune cells were discovered to be positively connected and are displayed in red, others were shown to be negatively related and are shown in blue. The cut-off was established at *p* < 0.05. **(B)** The genes expression data of uveal melanoma patients were divided into two clusters by ssGSEA analysis. **(C)** The PCA plot of distribution status of the two uveal melanoma clusters. **(D)** The heatmap showed that the 29 immune-related cells and types were enriched in the high immune cell infiltration group (Immunity_H), and low enrichment in the low immune cell infiltration group (Immunity_L). Using the ESTIMATE algorithm, each patient’s Tumor Purity, ESTIMATE Score, Immune Score, and Stromal Score were displayed with the clustering information. **(E)** The violin plot showed the difference in ESTIMATE Score, Immune Score, and Stromal Score between two clusters. **(F)** The box plot showed that there was a statistical difference in the expression of the HLA family between the two groups. **(G)** The gene functional enrichment analysis of Immunity_H and Immunity_L clusters. **(H)** Microenvironmental immune cell profiling of Immunity_L and Immunity_H. **p < 0.01, ***p < 0.001.

In the analysis, we obtained 80 UVM samples from the TCGA database. Then, the ssGSEA method was used to quantify the RNA-seq data of UVM samples to assess the infiltration level of 29 types of immune cells. The ssGSEA scores of each UVM sample were calculated and obtained. A heatmap was generated to illustrate the varied association patterns among the immune cell landscape in the tumor microenvironment (TME) (**Figure 1A**). According to the scores of ssGSEA, through an unsupervised hierarchical clustering algorithm, we found two clusters with different immune infiltration patterns, which include the Immunity_H cluster (n = 10) and Immunity_L cluster (n = 70) (**Figure 1B, C**). In order to validate the feasibility and practicability of the above grouping method, based on the expression profile of each UVM sample, the ESTIMATE algorithm was used to calculate tumor purity, Stromal Score, Immune Score, and ESTIMATE Score of two clusters. Compared with the Immunity_L cluster, the Stromal Score, Immune Score, and ESTIMATE Score were higher in the Immunity_H cluster, but Tumor Purity was the opposite (**Figure 1D**). The violin plot also has shown that Stromal Score, Immune Score, and ESTIMATE Score in the Immunity_H cluster become higher than those in the Immunity_L cluster (*p* < 0.001, **Figure 1E**). In addition, we also found that the expression of most of HLAs in the Immunity_H cluster becomes higher when compared with the Immunity_L cluster (all p < 0.05, **Figure 1F**).In KEGG analysis of genes between Immunity_H and Immunity_L clusters, various biological processes were enriched, such as natural killer cell-mediated cytotoxicity, T cell receptor signaling pathway, chemokine signaling pathway, B cell receptor signaling pathway, NOD-like receptor signaling pathway, Toll-like receptor signaling pathway, and so on (**Figure 1G**). More specifically, the Immunity_H cluster had significantly higher immune scores than the Immunity_L cluster of the 22 immune cell types tested, including APC cells, aDC cells, T cells, and Th1 cells (**Figure 1H**).

### 3.2 Identification of differentially expressed genes between immunity high and low clusters and immune-related genes

**Figure 2:**
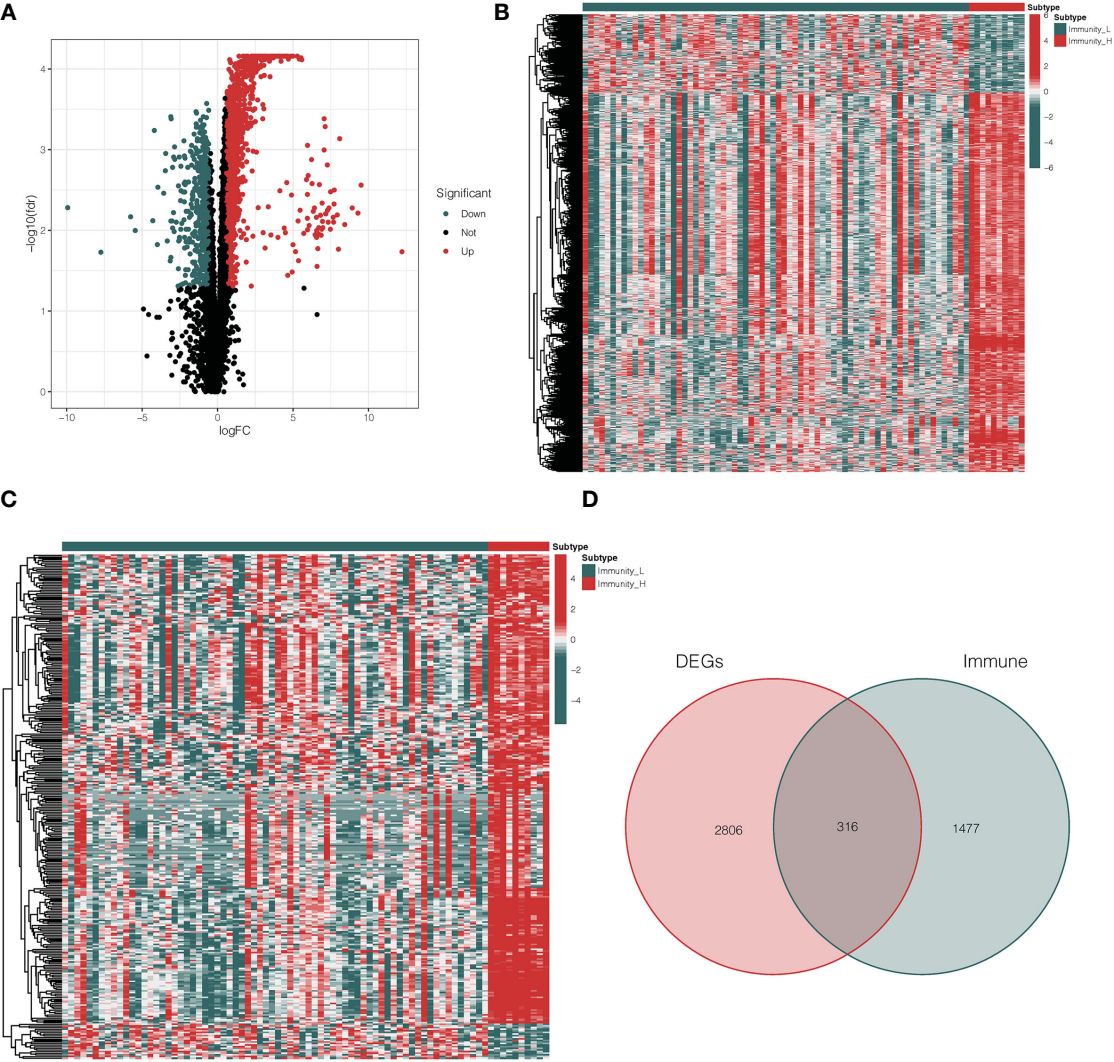
Analysis of differentially expressed immune-related genes. **(A)** The volcano plot showed that 2588 up-regulated genes and 534 down-regulated genes between Immunity_H and Immunity_L clusters. The red and blue bars stand for up-regulated genes and down-regulated genes, respectively. **(B)** The heatmap showed the expression level of DEGs in the Immunity_H and Immunity_L clusters. **(C)** The heatmap showed the expression level of immune-related genes in the Immunity_H and Immunity_L clusters. **(D)** The Venn diagram identified 316 genes from both gene sets. DEGs differentially expressed genes.

Based on a threshold of |logFC| > 1 and adj *p* < 0.05, we explored the DEGs between Immunity_H and Immunity_L clusters in the TCGA database. Then, we obtained 3122 DEGs including 2588 up-regulated genes and 534 down-regulated genes (**Figure 2A**, **Supplementary Table 2**). Heatmap showed the expression level of DEGs in the Immunity_H and Immunity_L clusters (**Figure 2B**). Besides, 2498 immune-related genes were downloaded from the ImmPort database (**Supplementary Table 3**). Heatmap showed the expression level of immune-related genes in the Immunity_H and Immunity_L clusters (**Figure 2C**). Furthermore, we performed a two-way Venn analysis based on the DEGs and immune-related genes from the ImmPort database, 316 genes were identified in both gene sets (**Figure 2D**, **Supplementary Table 4**).

### 3.3 Identification of immune-related genes prognostic signature for uveal melanoma

**Figure 3:**
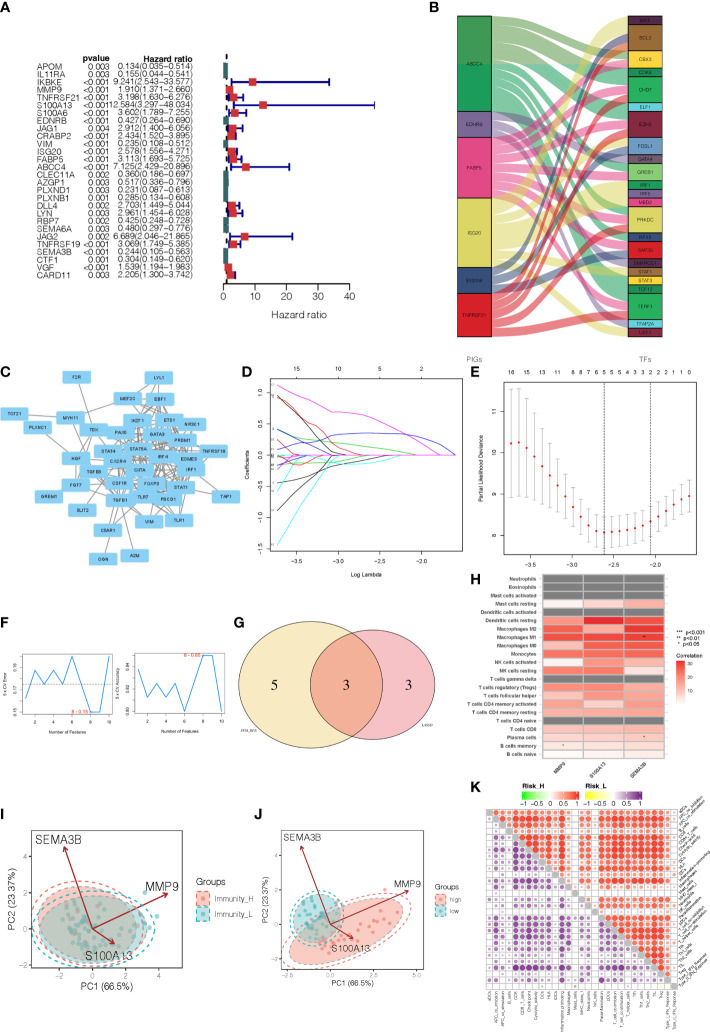
Identification of immune-related gene prognostic signature for uveal melanoma. **(A)** The HR and p-value from the selected genes in the immune terms using the univariable Cox regression analysis. **(B)** Alluvial diagram of the relationship between the 28 immune-related genes and transcription factors. **(C)** The interaction network of those immune-related prognostic genes. **(D)** The LASSO coefficient profiles of the 28 immune-related genes. **(E)** The optimal values of the penalty parameter were determined by 1,000-round cross-validation. **(F)** The accuracy and error of the estimate yield from SVM-RFE algorithm. **(G)** The common prognostic features were selected from the overlap of the LASSO and SVM-RFE algorithms. **(H)** The heatmap showed that the correlation between immune-related prognostic genes and immune infiltration cells. **(I)** PCA analyses for prognostic genes in two immunity level clusters. **(J)** PCA analyses for prognostic genes in high-risk and low-risk categories. **(K)** Microenvironmental immune cell profiling of high-risk and low-risk groups.

After integrating clinicopathological information into gene expression profiles, we obtained 80 UVM samples. In order to investigate the immune-related genes that could predict the prognosis of UVM, we performed univariate Cox regression analysis on the roles of 316 intersection genes. From this analysis, 28 genes were associated with OS according to the criterion of *p* < 0.001 (**Figure 3A**). In addition, to identify the correlation between 28 immune-related genes and transcription factors (TFs, **Supplementary Table 5**), we performed a correlation analysis. The alluvial diagram was developed to represent the relationship between the 28 immune-related genes and TFs (**Figure 3B**). Moreover, the interaction network of those genes was established *via* the STRING database and displayed by Cytoscape (**Figure 3C**). To narrow the prognostic signature, the LASSO regression algorithm on 28 immune-related genes and the optimal value of the parameter was identified by 1000-round cross-validation. The results of LASSO analysis indicated that the prognostic model achieved the best performance when it included three immune-related genes, including MMP9, S100A13, and SEMA3B (**Figure 3D, E**). Combining the genes eliminated by the LASSO and SVM-RFE algorithms led to the identification of 3 genes being chosen simultaneously by these two algorithms (**Figure 3F, G**), which were recognized as potential classification and prognosis characteristics. As shown in **Figure 3H**, MMP9 was correlated with B cells memory (p, < 0.05), and SEMA3B was correlated with macrophages M1 and plasma cells (*p* < 0.01 and *p* < 0.05, respectively). The dissemination of three prognostic genes screened from DEGs in various immunity clusters was validated using a PCA plot. This demonstrates that the training cohort’s prognostic genes were skewed in opposite directions between the Immunity_H and Immunity_L clusters (**Figure 3I**). A prognostic signature was constructed to calculate the risk score for each sample based on the expression level of these three genes. Risk score = (expression of MMP9 * 0.602435) + (expression of S100A13 * 1.333449) + (expression of SEMA3B * -1.092650). According to the median risk score, 80 UVM patients in the TCGA database were classified into a high-risk group and a low-risk group. This PCA plot shows that there are two directions in the distribution of our prognostic genes screened from DEGs, indicating low-risk and high-risk groups (**Figure 3J**). In addition, the high-risk group had significantly higher immune scores than the low-risk group of the 22 immune cell types tested, including APC cells, B cells, MHC cells, T cells, Th1 cells, and Th2 cells (**Figure 3K**). Three immune-related genes that are highly sensitive and specific prognostic indicators for UVM patients were discovered by these findings.

### 3.4 Validation of immune-related genes prognostic signature for uveal melanoma

**Figure 4:**
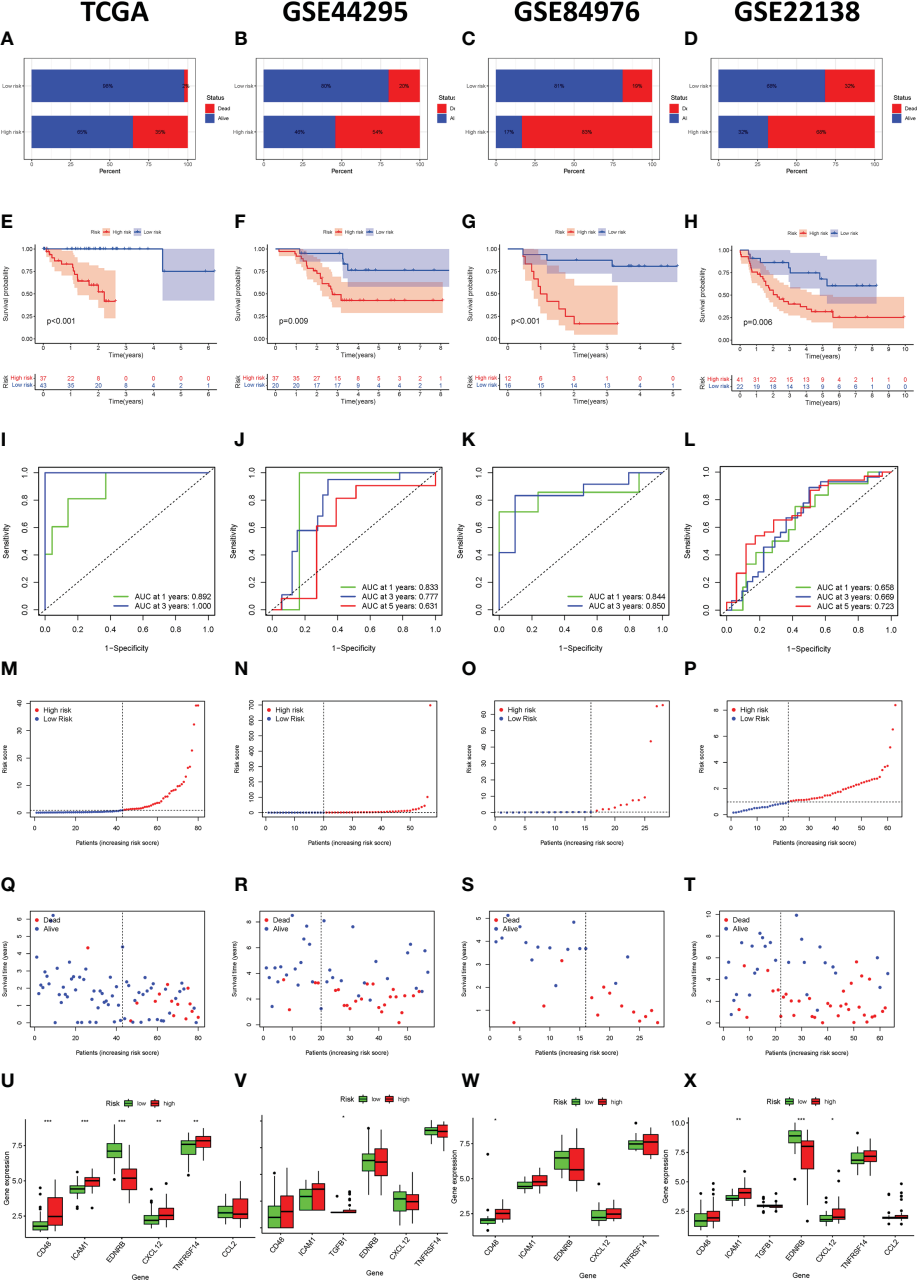
Immune-related gene prognostic signature used to predict outcomes in the TCGA set and GEO set. Survival status of patients in the high-risk and low-risk groups in the TCGA set **(A)**, GSE44295 set **(B)**, GSE84976 set **(C)**, and GSE22138 set **(D)**. The Kaplan-Meier survival curves for patients with uveal melanoma in the TCGA set **(E)**, GSE44295 set **(F)**, GSE84976 set **(G)**, and GSE22138 set **(H)**. The time-independent ROC curve of the prognostic signature at 1-year, 3-year, and 5-year in the TCGA set **(I)**, GSE44295 set **(J)**, GSE84976 set **(K)**, and GSE22138 set **(L)**. The risk curve of each uveal melanoma sample is reordered by risk score in the TCGA set **(M)**, GSE44295 set **(N)**, GSE84976 set **(O)**, and GSE22138 set **(P)**. The scatter plot of the uveal melanoma samples survival overview in the TCGA set **(Q)**, GSE44295 set **(R)**, GSE84976 set **(S)**, and GSE22138 set **(T)**. The immunosuppressive cytokine expression in the high-risk and the low-risk groups in the TCGA set **(U)**, GSE44295 set **(V)**, GSE84976 set **(W)**, and GSE22138 set **(X)**. *p < 0.05, **p < 0.01, ***p < 0.001.

The bar plot shows that the proportion of patients who died in the high-risk group was higher than those in the low-risk group (**Figure 4A**). The Kaplan-Meier survival curve and log-rank test indicated that patients in the high-risk group had a significantly worse OS than those in the low-risk group (**Figure 4E**). The areas under curve (AUC) value of the ROC curve for predicting the 1-year and 3-year OS rates were all higher than 0.8 (**Figure 4I**). The distribution of risk score and survival status of UVM samples were presented in **Figure 4M**, **5Q**. We also observed that immunosuppressive cytokines were also down-regulated in the low-risk group (**Figure 4U**).

**Figure 5:**
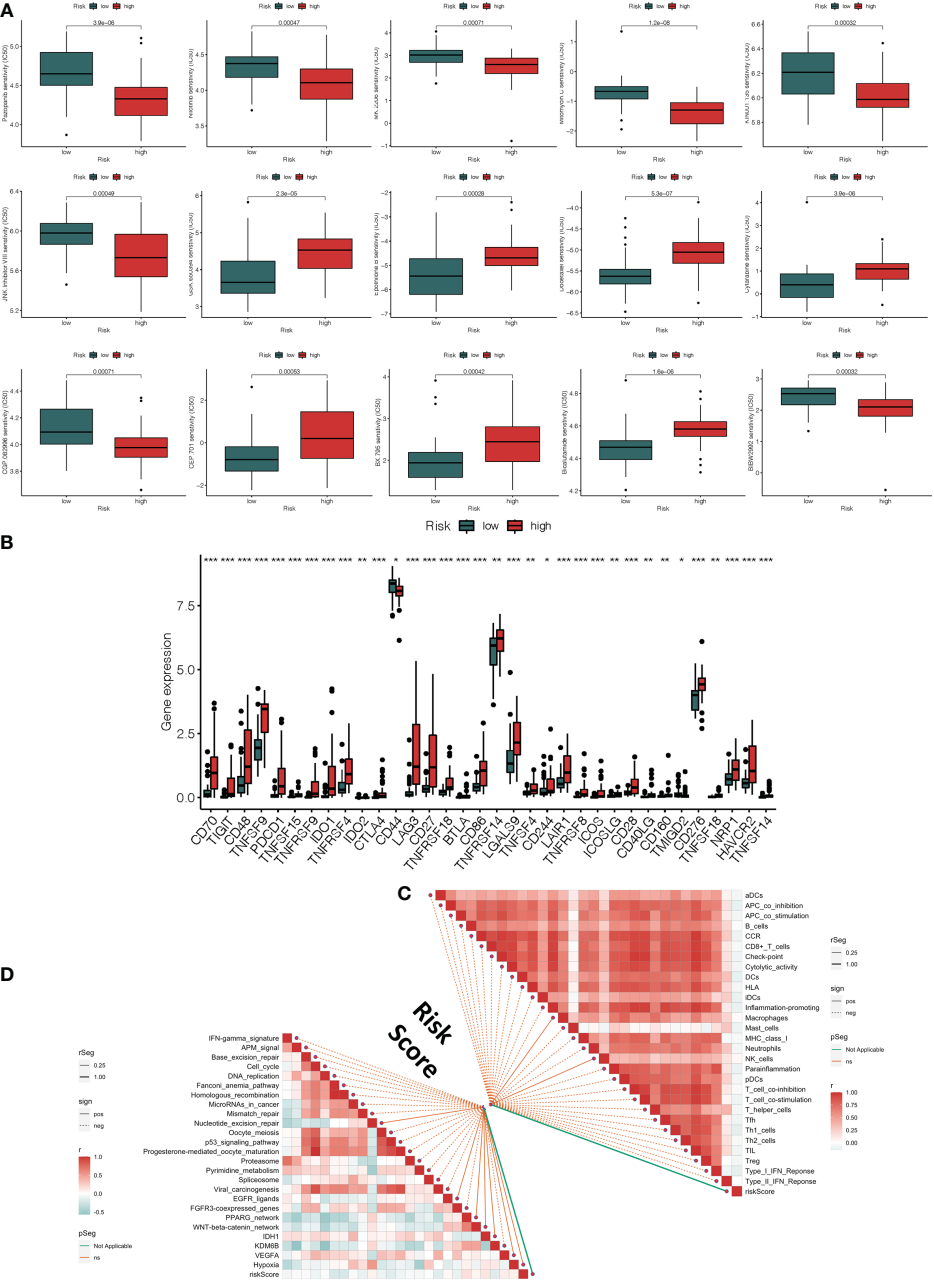
Clinical response to anti-tumor treatment and immune checkpoint-related gene expression in high and low-risk groups. **(A)** The prediction of chemotherapy and molecular medicines based on the risk groups **(B)** The difference in immune checkpoint expression in high-risk and low-risk groups. **(C)** Correlations between risk score and immune infiltrating cells. **(D)** Correlations between risk score and immunotherapy-predicted pathway enrichment scores. *p < 0.05, **p < 0.01, ***p < 0.001.

In order to verify the effectiveness and robustness of the immune-related genes prognostic signature in predicting the OS of UVM patients. The GSE44295 GSE84976 database including 57 and 28 UVM samples were used as the validation set. According to the above immune-related prognostic model, we divided patients in the validation set into high-risk and low-risk groups based on the median risk score. As shown in **Figure 4B, C**, patients in the high-risk group had higher mortality rates than those in the low-risk group. The Kaplan–Meier survival curve showed that the survival outcome of the high-risk group was worse than those in the low-risk group (p = 0.009 and *p* < 0.001, **Figure 4F, G**). In addition, the ROC curve showed that the AUCs of risk scores for predicting the 1-year, 3-year, and 5-year survival rates in the GSE44295 were 0.833, 0.777, and 0.631, respectively (**Figure 4J**). The AUC was 0.844 for 1-years and 0.850 for 3-years in the GSE84976 cohort (**Figure 4K**). Besides, the risk score and survival status of the prognostic signature were shown in the scatter plot (**Figure 4N, O, R, S**). We also observed that immunosuppressive cytokines were also down-regulated in the low-risk group (**Figure 4V, W**). Our results suggest that the risk score was a good model for predicting the OS of UVM patients.

Furthermore, we also analyzed the effectiveness and robustness of the immune-related genes’ prognostic signature in predicting the metastasis-free survival (MFS) of UVM patients. The GSE22138 database including 63 UVM samples were used as independent validation sets. Similarly, patients in the high-risk group had higher mortality rates than those in the low-risk group (**Figure 4D**). The Kaplan-Meier survival curve and log-rank test indicated that high-risk patients had significantly shorter MFS rates than those high-risk patients (p = 0.006, **Figure 4H**). The AUC of the ROC curve for predicting the 1-year, 3-year, and 5-year OS rates were 0.658, 0.669, and 0.723, respectively (**Figure 4L**). Besides, the risk score and survival status of prognostic signature were shown in the scatter plot (**Figure 4P, T**). We also observed that immunosuppressive cytokines were also down-regulated in the low-risk group (**Figure 4X**). Our results suggest that the risk score was a good model for predicting the MFS of UVM patients.

### 3.5 Analysis of clinical response to anti-tumor treatment, immune status, and immune microenvironment

Using the R package “pRRophetic,” we explored the link between risk score and the clinical response to several immunotherapeutic medications as well as some chemotherapy medicines. The sensitivity to top 15 chemical or targeted therapies between the high-risk and low-risk groups was significantly different as measured by the half-maximal inhibitory concentration (IC50) of anti-tumor medications (**Figure 5A**). The majority of immunological checkpoints were more active in the high-risk group. Additionally, we discovered that several immune checkpoint genes of immunotherapy, such as the increase in CD70, CD48, CTLA4, and TNFSF4 gene expression in the high-risk group, revealed that they had different impacts in each group (**Figure 5B**). It suggested that based on risk score, we might choose the best checkpoint inhibitors for UVM patients. Based on the CIBERSOFT algorithms, we found a positive correlation between risk score and the levels of immune cell infiltration of the CD4 T cell, B cell, NK cell, dendritic cell, and macrophage (**Figure 5C**). We also evaluated the relationships between risk score and immune pathways for immunotherapy, such as oncogenic pathways, gene signatures linked with targeted therapy, and radiation response gene signatures (**Supplementary Table 6**). Approximately all markers connected to anticancer immunotherapy have a positive correlation between the risk score and the enrichment scores (**Figure 5D**).

### 3.6 Evaluation of TMB, prognostic factor, and the nomogram of the immune-related gene prognostic signature

**Figure 6:**
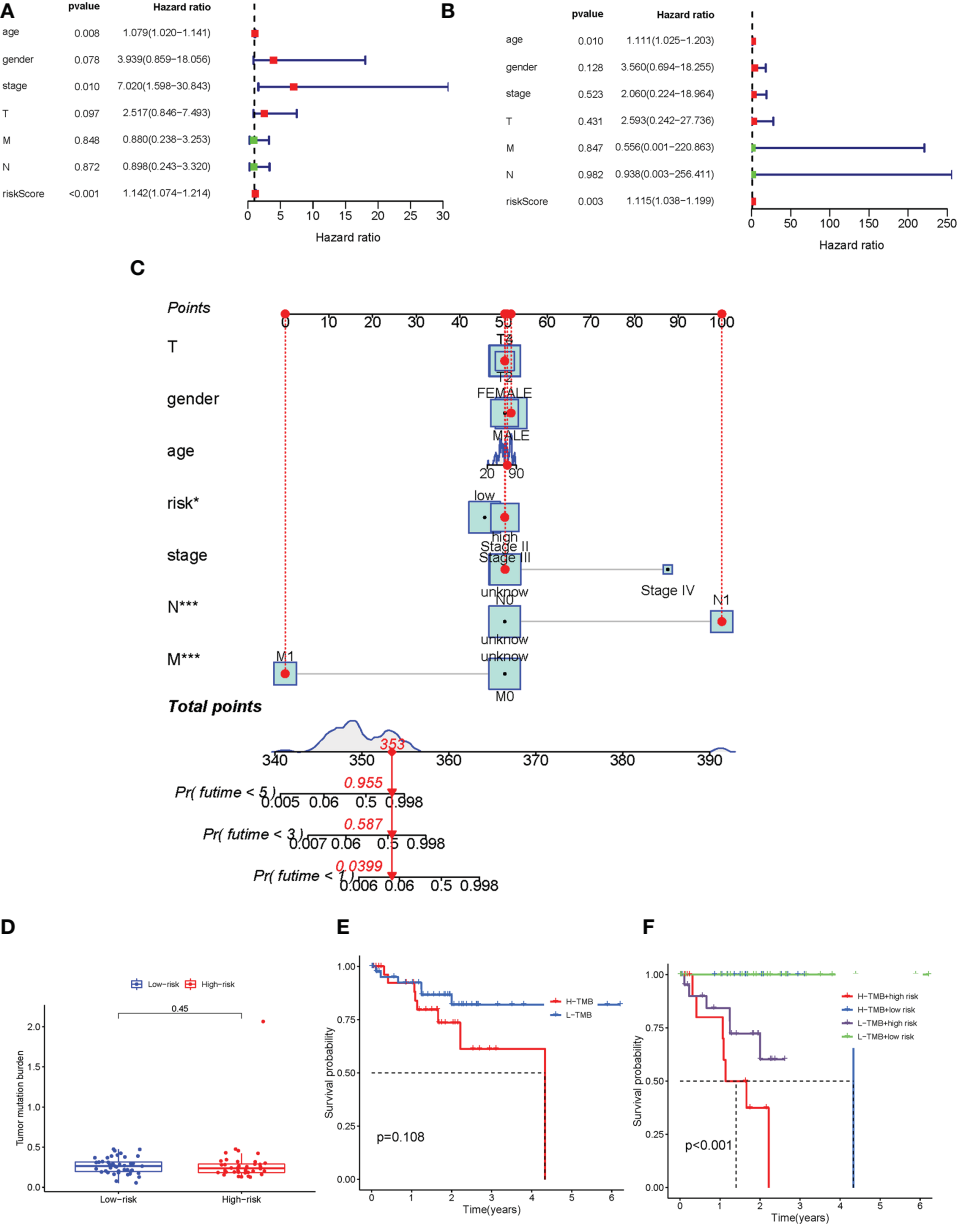
Evaluation TMB, prognostic factor, and the nomogram of the immune-related gene prognostic signature. **(A-B)** Univariate **(A)** and multivariate **(B)** Cox regression analysis evaluating the independent risk factors of the immune-related gene prognostic signature in uveal melanoma patients. **(C)** Development of a nomogram based on the immune-related gene prognostic signature in the TCGA training cohort. **(D)** The box plot for TMB levels for patients in the high-risk and low-risk groups. **(E)** Kaplan–Meier curves for the high-TMB and low-TMB of uveal melanoma patients. Log-rank test shows overall p = 0.108. **(F)** Kaplan–Meier curves for uveal melanoma patients in TMB status and combined in the high-risk and low-risk groups. Log-rank test shows overall *p* < 0.001. TMB, tumor mutational burden. *p < 0.05 and ***p < 0.001.

The univariate and multivariate Cox regression analyses were performed to assess whether the immune-related genes prognostic model can be regarded as an independent predictor. The above results indicated that the novel prognostic model could work as an independent prognostic factor related to the survival outcome of UVM patients (**Figure 6A, B**). In order to predict the 1-year, 3-year, and 5-year OS rate of UVM patients a nomogram was established based on the TCGA database. We selected age, gender, stage, TMN status, and risk score were used as variables (**Figure 6C**).

In order to identify the potential correlation between immune-related gene prognostic signature and tumor mutational burden (TMB), we analyzed the TMB level of high-risk and low-risk groups (**Supplementary Table 7**). Our results showed that there was no significant difference in TMB levels between high-risk and low-risk groups (**Figure 6D**). The Kaplan-Meier survival analysis also indicated that the patients in the high-risk group had no significant difference in OS probability from those in the low-risk group (**Figure 6E**). We next analyzed the correlation of immune-related genes’ prognostic signature with TMB. The survival analysis was conducted on the risk group to determine the role of TMB status, which were low-risk group/low-TMB, low-risk group/high-TMB, high-risk group/low-TMB, and high-risk group/low-TMB. As shown in **Figure 6F**, there was a significant difference between those four groups (*p* < 0.001). In aggregate, these results suggested that risk score was correlated with somatic mutation patterns.

### 3.7 Single-cell sequencing data analysis

**Figure 7:**
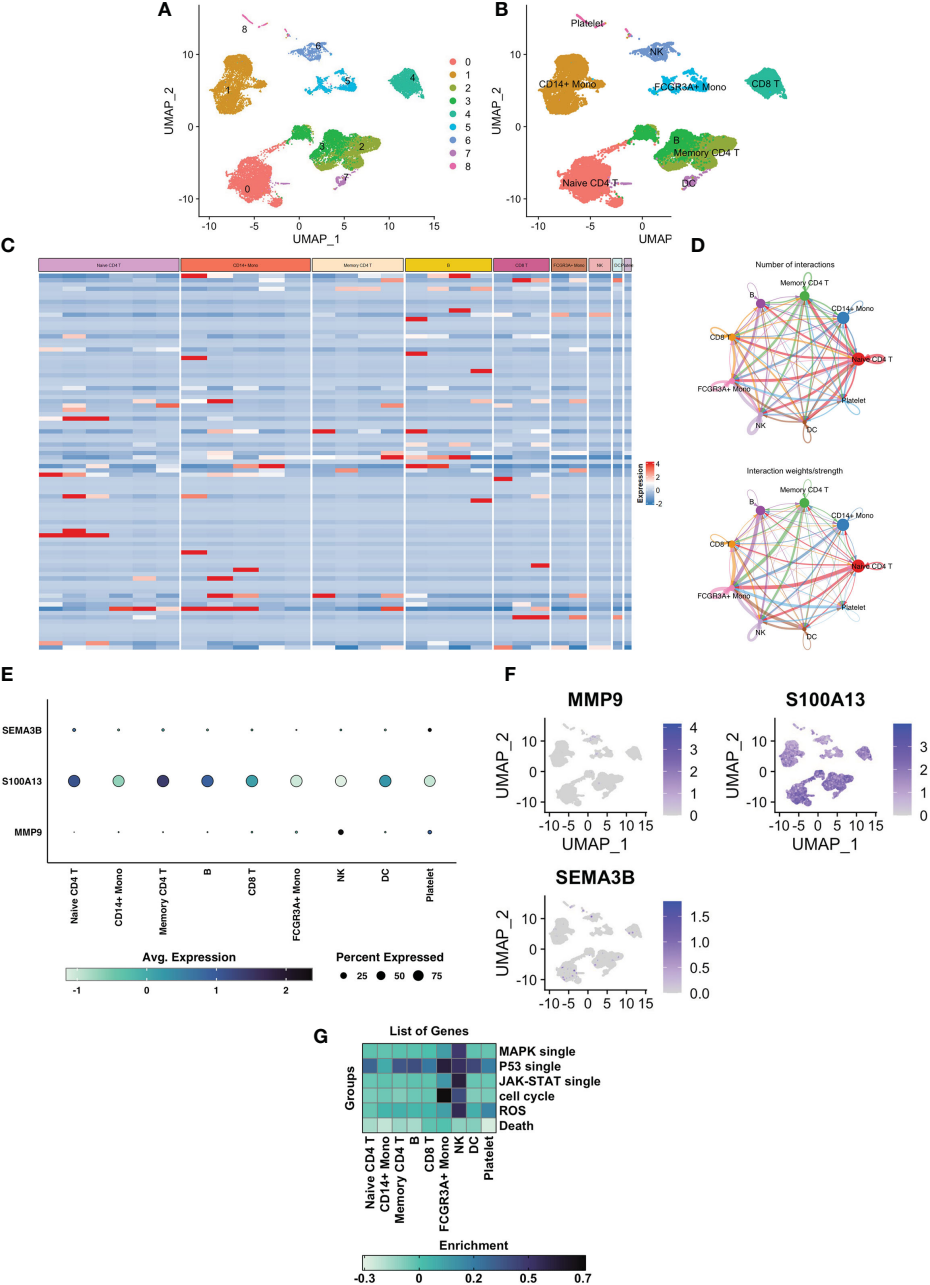
Single cell sequencing analysis of GSE139829 and the cell localization of 3 modelling genes. **(A)** Cluster analysis and dimension reduction. All of the cells in GSE139829 were divided into 9 cell clusters. **(B)** The cells are classified as Naive CD4+ T cells, CD14+ Mono cells, Memory CD4+ cells, B cells, CD8+ T cells, Mono cells, NK cells, DC cells, platelet cells based on surface marker genes. **(C)** Heatmap shows characteristics that are differently expressed in each cell type. **(D)** A net plot displaying the quantity and intensity of interactions. **(E)** The three prognostic genes expression levels in the 8 cell types. **(F)** UMAP plots of the three prognostic genes. **(G)** Heatmap plot showing the correlation between specific signaling pathways and cell clusters.

After the application of a quality control standard, we used a UMAP method to decrease the dimensionality of these datasets in order to illustrate the distribution of the single-cell sequencing profile (**Figure 7A**). Cells were effectively categorized by unsupervised classification into 9 clusters (**Figure 7B, C**). We manually annotated these clusters as the following 9 cell types based on the CellMarker markers’ expression patterns: 1) Naïve CD4+ T cells (expressing IL7R and CCR7); 2) CD14+ Mono cells (expressing CD14 and LYZ); 3) Memory CD4+ cells (expressing IL7R and S100A4); 4) B cells (expressing MS4A1); 5) CD8+ T cells (expressing CD8A); 6) Mono cells (expressing FCGR3A, MS4A7, and FCGR3A); 7) NK cells (expressing GNLY, and NKG7); 8) DC cells (expressing FCER1A and CST3); 9) platelet cells (expressing PPBP). Then we began searching into how mural cells communicate with immune cells differently in UVM. We used the CellcRhat package to appreciate intracellular interactions by predicting protein-protein interactions using an existing database. The net plot revealed that CD4+ T cells and CD8+ T had the most interactions of any cell type (**Figure 7D**). We used single-cell sequencing data to investigate how modeling genes are expressed in various cell types. MMP9 was predominantly expressed in NK cells, SEMA3B was predominantly expressed in Naive CD4+ T cells and platelet cells, and S100A13 was predominantly expressed in Naive CD4+ T cells, Memory CD4+ T cells, and CD8+ T (**Figure 7E, F**). Finally, we investigated the correlation between specific signaling pathways and cell clusters. We found that the ROS pathway is mainly enriched in NK cells and platelet cells (**Figure 7G**).

### 3.8 Experiment validation *in vitro* and *ex vivo*

To further confirm the expression pattern of MMP9, S100A13, and SEMA3B, the mRNA and protein expression levels of MMP9, S100A13, and SEMA3B were analyzed in the UVM cell line (C918) and normal adult retinal pigmental epithelial cell line (ARPE-19; **Supplementary Figure 1A**). Our results showed that the mRNA expression level of MMP9, S100A13, and SEMA3B was increased in the C918 cell line (**Supplementary Figure 1B**). The protein expression level of MMP9, S100A13, and SEMA3B were also investigated between the UVM cell line and the normal cell line (Supplementary **Figure 1C**).

**Figure 8:**
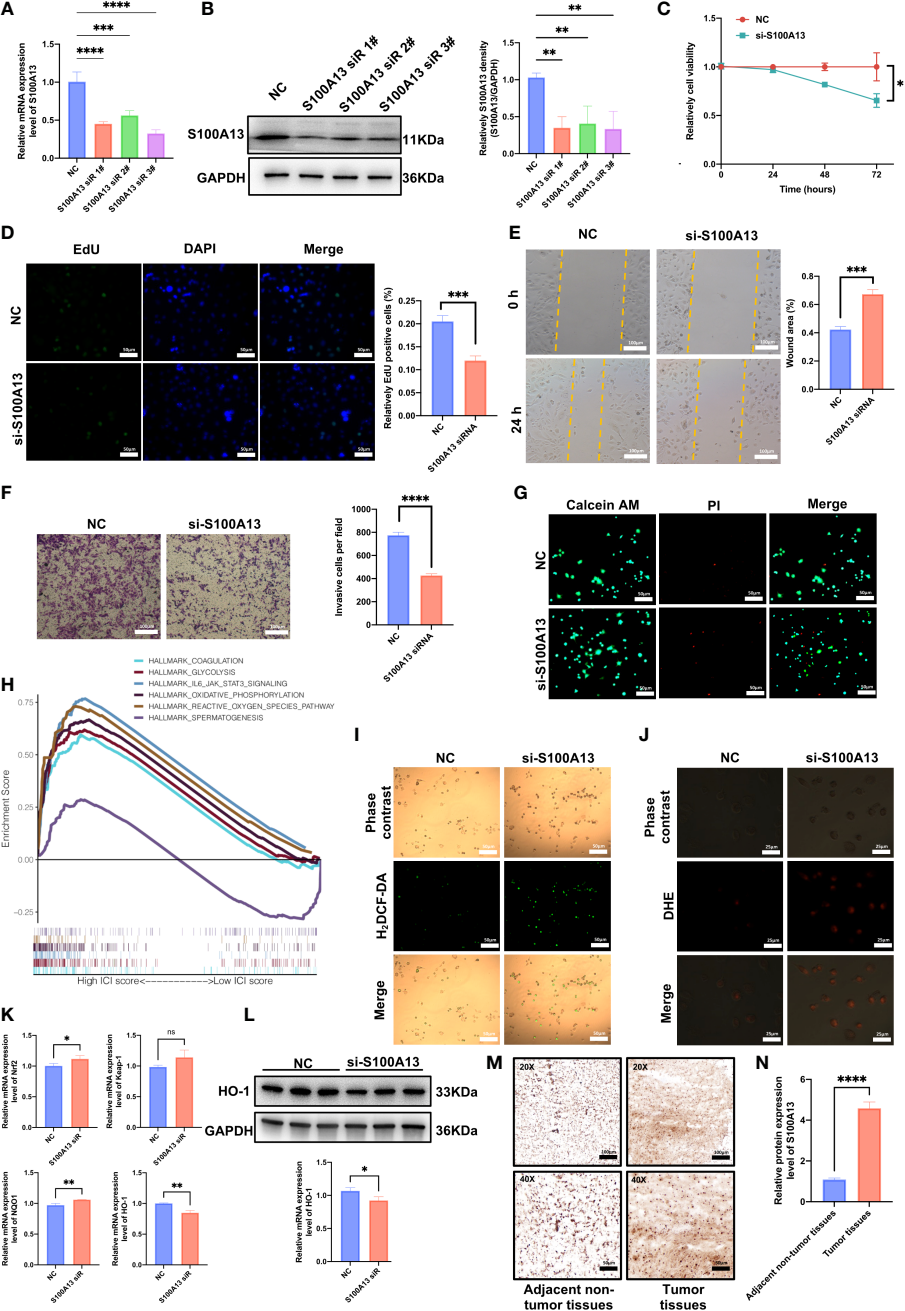
S100A13 inhibits the proliferation and migration capacity of UVM cells by disturbing the level of oxidative stress. **(A)** The qRT-PCR assays validated the siRNA knockdown effect. **(B)** Western blot experiment validated the siRNA knockdown effect with statistical analysis. **(C)** The results of CCK-8 assay. **(D)** EdU assay of UVM cell lines treated with siRNA or negative control (NC) of S100A13. **(E)** Scratch wound healing assay of UVM cell lines treated with siRNA or NC of S100A13. **(F)** Transwell assay of UVM cell lines treated with siRNA or NC of S100A13. **(G)** The live/dead staining assay of UVM cell lines treated with siRNA or NC of S100A13. **(H)** According to the GSEA results, there is a significant positive correlation between S100A13 expression and the ROS pathway. **(I)** H_2_DCF-DA staining of UVM cell lines treated with siRNA or NC of S100A13. **(J)** DHE staining of UVM cell lines treated with siRNA or NC of S100A13. **(K)** The mRNA expression of ROS-related gens in UVM cell lines treated with siRNA or NC of S100A13. **(L)** Western blot experiment validated the protein level of HO-1 based on siRNA knockdown effect with statistical analysis. **(M)** Illustrations of S100A13 immunohistochemistry (IHC) staining in tumor tissues and adjacent non-tumor tissues. **(N)** Relative expression of the S100A13 in tumor tissues and adjacent non-tumor tissues. *p < 0.05, **p < 0.01, ***p < 0.001, ****p < 0.0001; ns, no significance.

In order to confirm the role of certain genes in C918 cell proliferation and migration, we further did the experimental investigation on prognostic markers. The oncogenic effect of S100A13 was examined in additional experiments since gene S100A13 had a very high coefficient value and was resilient in the earlier-built models. We then carried out loss-of-function experiments that silence S100A13 in the C918 cells to examine the biological role of S100A13 in UVM progression. The qRT-PCR and WB validated the effectiveness of the siRNA knockdown in C918 cells, and si-S100A13-3 was chosen for the following tests since it demonstrated the highest level of gene silencing effectiveness (**Figure 8A, B**). The CCK-8 assay revealed that after the S100A13 knockdown, the cells’ viability was significantly reduced in 72 hours (**Figure 8C**). EdU staining assay was also used in the assessment of cell proliferation. Cells with a reduced S100A13 expression exhibited a significant decrease in the number of EdU positive cells, compared with the siRNA negative control group (**Figure 8D**). In addition, transwell invasion and scratch assay results showed that UVM cell migration and invasion were drastically reduced when S100A13 was knocked down (**Figure 8E, F**). In line with the CCK-8 results, the live/dead staining assay results of all the groups reveal that the number of dead C918 cells drastically increased when S100A13 was knocked down (**Figure 8G**). These findings suggest that the S100A13 gene may play an important role in UVM cell survival. According to the GSEA results, S100A13 expression is significantly and positively correlated with the ROS pathway signatures (**Figure 8H**). Oxidative stress plays an important role in UVM. Fluorescence staining revealed that ROS production was significantly increased after S100A13 knockdown in the C918 cells (**Figure 8I**). Meanwhile, by fluorescence staining, DHE was found to be unexpectedly up-regulated in S100A13 knockdown C918 cells compared to controls (**Figure 8J**). Furthermore, we also evaluated ROS-related gene expression. qRT-PCT revealed that Nrf2 and NQO1 expression was up-regulated in the S100A13 knockdown C918 cells compared to control (**Figure 8K**), whereas HO-1 expression was down-regulated as detected by Western blot (**Figure 8L**). These results indicated S100A13 might act a part in UVM progression by alleviating oxidative stress.

In order to identify the representative S100A13 protein levels in UVM tissue and adjacent non-tumor tissues acquired from patients being treated at the Eye Center of Xiangya Hospital, immunohistochemistry (IHC) labeling was performed. In line with the earlier findings, UVM tissue had much higher S100A13 protein levels than adjacent non-tumor tissues (**Figure 8M, N**).

## 4 Discussion

UVM is one of the most common ocular malignant tumors, with highly heterogeneous genetic patterns and poor prognosis. In this respect, early detection, diagnosis, and treatment may bring clinical benefits to patients with a UVM prognosis. So, there is an urgent need to find an effective and robust prognostic biomarker for UVM patients. UVM is a complex and heterogeneous malignant tumor characterized by multiple genetic mutations and immune cell infiltration. Therefore, accumulative studies focused on tumor immunity microenvironment, immunotherapy is of great significance for improving the survival for a wide variety of cancers, including metastatic melanoma (29–31). Previous studies have shown that immune infiltration patterns such as immune and stromal cells are related to the prognosis of UVM patients (32, 33). Currently, the whole-genome transcriptomics studies insights on cancer have suggested that immune-related genes can be used to help determine the survival outcome of patients with cancer or responses to individual immunotherapies (34).

**Figure 9:**
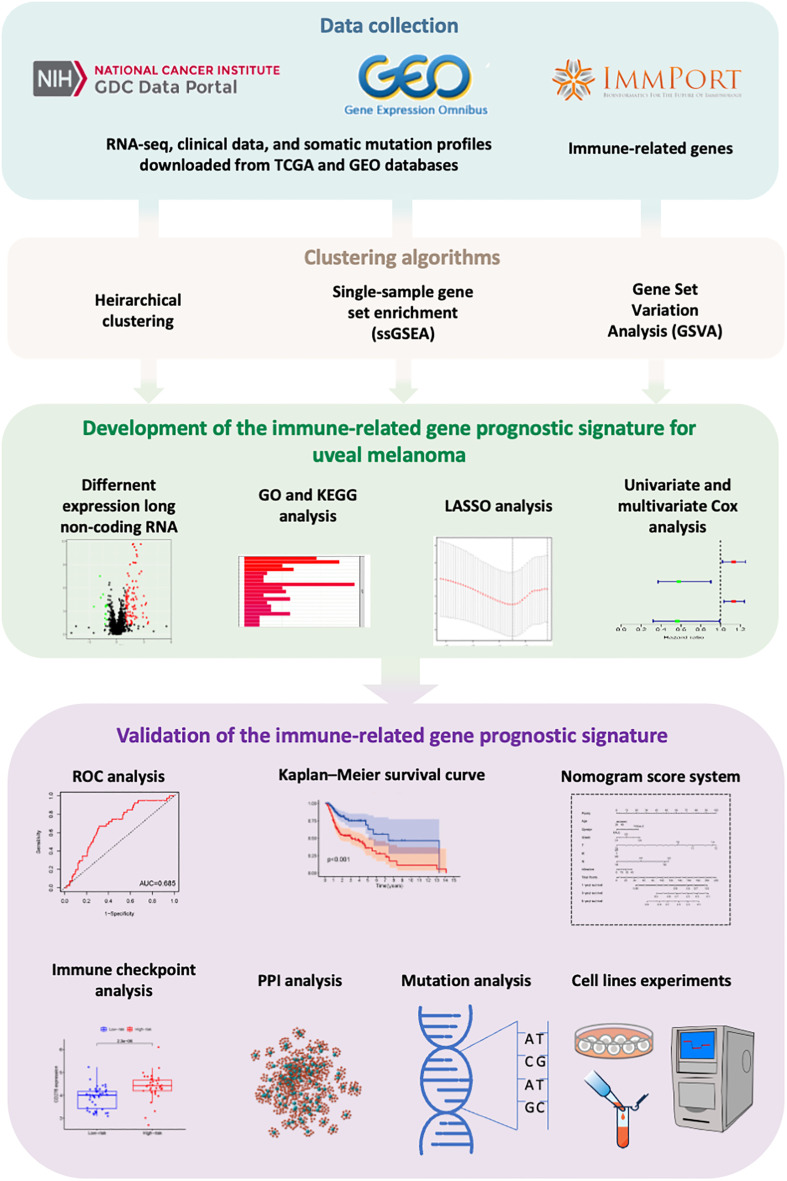
The workflow of this study.

In this study, we constructed a gene signature with prognostic values related to immunity. Firstly, ssGSEA was used to group UVM patients into an Immunity_H and Immunity_L cluster based on the infiltration level of 29 types of immune cells. Then, the ESTIMATE and CIBERSORT algorithms were used to validate the results of the ssGSEA method and reflect the infiltration levels of immune cells and stromal cells between Immunity_H and Immunity_L clusters. We, using univariate Cox regression analysis, identified 14 immune-related DEGs in both immunity clusters and the ImmPort database, which were significantly associated with the OS of patients with UVM. Next, LASSO, SVM-RFE algorithm, and multivariate Cox regression analyses, hub genes (including MMP9, S100A13, and SEMA3B) with significantly prognostic values from the immune-related genes were identified (**Figure 9**).

Furthermore, the Kaplan-Meier survival analysis and ROC curve were used to confirm that this three-gene signature is an effective and robust prognostic predictor of OS for patients with UVM in the training set. According to the prognostic model, the risk score of each patient in the TCGA cohort was calculated and UVM patients were divided into low- and high-risk subgroups based on the median risk score. We found that patients in the high-risk group showed markedly poorer OS than those in the low-risk group. Additionally, the prognostic signature was also proved to be an effective model for predicting OS in the GSE84976 and GSE44295 cohorts and MFS in the GSE22138 cohort. After identifying the prognostic model, we also validated the three-genes prognostic signature in two independent validation sets using the Kaplan-Meier survival analysis and ROC curve. Moreover, a nomogram was established based on the three-gene prognostic signature that can be used to predict the prognosis of UVM patients.

At present, the therapeutic benefits of immune checkpoint inhibitors in the occurrence and advancement of tumors have been widely recognized. Immune checkpoint treatment has a limited impact on UVM (35). Retrospective statistics indicate that the response rate is quite low (36). We analyzed the relationship between risk score and the expression of immune checkpoint genes. Our results have shown that the risk model was positively correlated with immune checkpoint genes (CD27, CD28, CD276, and LAG3) expression levels. In addition, immune checkpoint gene expression levels were higher in the high-risk group than those in the low-risk group. Compared with cutaneous melanoma patients, UVM, especially metastatic UVM, showed a poor efficacy of immune checkpoint blockade. A clinical study of Ipilimumab, a monoclonal antibody targeting cytotoxic T-lymphocyte-associated antigen 4 (CTLA-4), in patients with UVM obtained a median OS of 5.2-10.3 months (37). Pembrolizumab, a humanized monoclonal anti-PD1 antibody, has shown a 20% response rate with a median progression-free survival at 11 months (36). GNAQ or GNA11 mutations with low TMB frequently occur in UVM patients, nearly 80~93% (38). Epithelioid cell type, monosomy 3 and 6p gain, and deletion of the BAP-1 gene are among the histopathologic and tumor-specific genetic abnormalities that are most important for melanoma-specific mortality prediction (3). UVM shows a lower TMB than cutaneous melanoma, which is related to the weak efficiency of immunotherapy (1, 39).

The TME landscape shows that the immune cell compositions of the two groups differ significantly, which may aid researchers in creating a novel or more efficient therapeutic options for enhancing immune responses. While APC cells and DCs were more abundant in the high-risk group, macrophage cells were more enriched in the low-risk group. Through a variety of ways, including by influencing immune and nonimmune cells inside or outside of the tumors, Tregs maintain tumor immune exclusion. In UVM, the immunosuppressive behavior of Tregs is thought to be a major impediment to efficient antitumor immunity. macrophages are crucial in the production of inflammatory cytokines and the activation of the immune system. Our immune-related gene prognostic signature will provide new information for personalized cancer immunotherapy and improve clinical outcomes.

Furthermore, three genes (MMP9, S100A13, and SEMA3B) were selected as crucial immune-related prognostic signatures. MMP9 is an important member of the zinc-dependent endopeptidases family. In particular, MMP9 belongs to the gelatinase subgroup. Some studies have reported that MMP9 is one of the most widely investigated MMPs, which plays a vital role in proteolytic degradation of the extracellular environment. Because of its biological role, MMP9 is involved in tumor cell invasion and cancer cell metastasis (40). A previous study also proved that the overexpression of MMP9 might contribute to increasing breast cancer cell line malignancy through modulation of the transforming growth factor-beta/SMAD signaling pathway (41). Li et al. reported that the knockdown of MMP9 could suppress the *in vitro* angiogenesis ability of cutaneous melanoma cell lines (42). In addition, MMP9 plays a crucial role in melanoma cell metastasis and invasion through CD147/NFAT1/MMP9 pathway (43). S100A13, a member of the S100 family, plays an important role in the release of fibroblast growth factor-1 and IL-1 (44, 45). The fibroblast growth factor-1 and IL-1 are known to be involved in angiogenesis, inflammation, and tumor metastasis (46). Miao et al. reported that S100A13 is upregulated in human non‐small cell lung cancer, where it correlates with intratumoral angiogenesis, and is also associated with poor prognosis in patients with lung cancer (47). In addition, there is also a significant correlation between S100A13 expression and the survival duration of patients with melanoma (48). Several studies reported that S100A13 is also involved in the cell cycle progression and differentiation of melanoma tumors (49, 50). SEMA3B, a member of the semaphore’s family of soluble proteins, has been involved in several biological processes such as cell proliferation, apoptosis, and migration (51). Previous studies showed that SEMA3B is characterized as a strong tumor-suppressing factor in various cancers (52, 53). Guo et al. reported that the expression level of SEMA3B is frequently lower in gastric cancer cells and tissue, due to the fact, that the overexpression of SEMA3B and SEMA3B-AS1 could inhibit gastric cancer cell proliferation, migration, and invasion *in vitro (* 54). In addition, cisplatin could restore the homeostasis of endometrial cancer cells and improve the effectiveness of pharmacotherapy by increasing the expression of SEMA3B *in vitro (* 55). Recently, Hou et al. reported a 10-genes DNA methylation-driven signature including SEMA3B (56). However, the biological mechanism behind SEMA3B expression and the prognosis and initiation of malignant melanoma is not clear yet. Consistently, our cell experiment found that compared with normal retinal pigmental epithelial cells, the mRNA and protein level of MMP9, S100A13, and SEMA3B in the UVM cell line was up-regulated. To confirm the role of S100A13 in the UVM, we carried out a series of *in vitro* experiments. Our findings suggest that the S100A13 play an important role in UVM tumorigenesis by modulating cell viability and proliferation.

Our study identified an immune-related gene prognostic signature by using a serial bioinformatics strategy, it was not only validated in three relatively large independent patient cohorts but also exhibited significant clinical value and reliable performance in the prediction of patients’ survival outcomes. We also constructed the major immune-related genes-TFs network to reveal the possible mechanism in immune gene regulation. Additionally, the multivariate Cox prediction model is established for identifying the prognostic independent factors to predict UVM prognosis. The novel immune-related gene prognostic signature provides a prognosis assessment for UVM patients.

## Data availability statement

The original contributions presented in the study are included in the article/**Supplementary Material**. Further inquiries can be directed to the corresponding author.

## Author contributions

Conceptualization: WW and SW; methodology: WW; software: WW; validation: WW and HZ; formal analysis: WW; investigation: WW; resources: WW and HZ; data curation: WW and HZ; writing—original draft preparation: WW and SW; writing—review and editing: WW and SW; visualization: WW and HZ; supervision: SW; project administration: WW and SW; funding acquisition: WW and SW. All authors contributed to the article and approved the submitted version.

## Supplementary material

Supplementary Table 2 The DEGs between Immunity_H and Immunity_L clusters in the TCGA database.

Supplementary Table 3 Immune-related genes list from Import database.

Supplementary Table 4 The intersection gene between the immune-related gene and DEGs.

Supplementary Table 5 The transcription factors list.

Supplementary Table 6 The details of gene signatures in predicting radiotherapy responses.

Supplementary Table 7 Tumor mutational burden information of TCGA-UVM cohort.
