# Supplementary material for: Identification of a novel immune-related gene signature for prognosis and the tumor microenvironment in patients with uveal melanoma combining single-cell and bulk sequencing data
Source: Front Immunol. 2023 Jan 30;14:1099071. doi: 10.3389/fimmu.2023.1099071 (PMC9922847; doi:10.3389/fimmu.2023.1099071)
Supplement: Supplementary file 1 [file DataSheet_1.docx]

Supplementary Material

# Supplementary Data

## Supplementary Figures and Tables


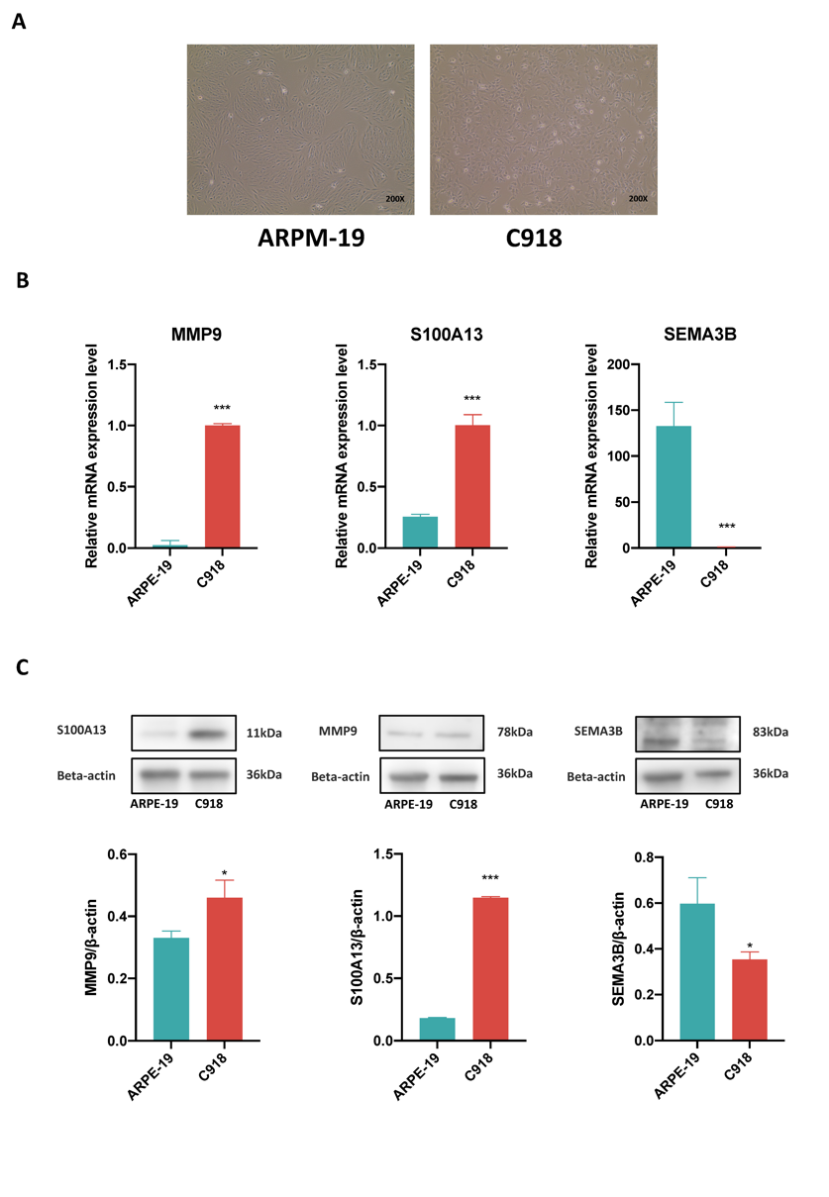


**Supplementary Figure 1.** Validation of the expression of the immune-related gene prognostic signature in UVM cell lines. (A) The representative figure of ARPE-19 and C918 cell lines. (B) The mRNA expression level of MMP9, S100A13, and SEMA3B in ARPE-19 and C918 cell lines. (C) The protein expression level of MMP9, S100A13, and SEMA3B in ARPE-19 and C918 cell lines. **p* < 0.05, ***p* < 0.01, ****p* < 0.001.

**Supplemental Table S1.** The clinical and pathological characteristics of uveal melanoma patients in the TCGA-UVM, GSE22138, GSE84976, and GSE44295 cohorts.

| **Characteristics** | **Training Cohort**  **(TCGA-UVM, 80 Cases)** | **Validation Cohort**  **(GSE22138, 63 Cases)** | **Validation Cohort**  **(GSE84976, 28 Cases)** | **Validation Cohort**  **(GSE44295, 57 Cases)** |
| --- | --- | --- | --- | --- |
| Age at diagnosis, years |  |  |  |  |
| <65 | 45(56.25%) | 36(57.14%) | 12(42.86%) | NA |
| ≥65 | 35(43.75%) | 27(42.86%) | 16(57.14%) | NA |
| gender |  |  |  |  |
| female | 35(43.75%) | 24(38.1%) | NA | 25(43.86%) |
| male | 45(56.25%) | 39(61.9%) | NA | 32(56.14%) |
| T classification |  |  |  |  |
| T1 | 0 | NA | NA | NA |
| T2 | 4(5%) | NA | NA | NA |
| T3 | 36(45%) | NA | NA | NA |
| T4 | 38(47.5%) | NA | NA | NA |
| unknown | 2(2.5%) | NA | NA | NA |
| M classification |  |  |  |  |
| M0 | 73(91.25%) | 28(44.44%) | NA | NA |
| M1 | 3(3.75%) | 35(55.56%) | NA | NA |
| unknown | 4(5%) | 0 | NA | NA |
| N classification |  |  |  |  |
| N0 | 76 (95.00%) | NA | NA | NA |
| N1 | 0 (0.00%) | NA | NA | NA |
| unknown | 4 (5.00%) | NA | NA | NA |
| tumor stage |  |  |  |  |
| stage I | 0 | NA | NA | NA |
| stage II | 36(45%) | NA | NA | NA |
| stage III | 40(50%) | NA | NA | NA |
| stage IV | 4(5%) | NA | NA | NA |
| tumor eye side |  |  |  |  |
| left | NA | 33(52.38%) | NA | NA |
| right | NA | 30(47.62%) | NA | NA |
| tumor cell type |  |  |  |  |
| epithelioid | NA | 21(33.33%) | NA | NA |
| mixed | NA | 23(36.51%) | NA | NA |
| unknown | NA | 19(30.16%) | NA | NA |
| tissue or organ of origin diagnosis |  |  |  |  |
| choroid | 67(83.75%) | NA | NA | NA |
| ciliary body | 5(6.25%) | NA | NA | NA |
| overlapping lesion of eye and adnexa | 8(10%) | NA | NA | NA |

TCGA: The Cancer Genome Atlas; UVM: Uveal Melanoma; NA: data not available.
